# On the interrelation between alcohol addiction–like behaviors in rats

**DOI:** 10.1007/s00213-021-06059-4

**Published:** 2022-01-12

**Authors:** Johanna A. S. Smeets, A. Maryse Minnaard, Geert M. J. Ramakers, Roger A. H. Adan, Louk J. M. J. Vanderschuren, Heidi M. B. Lesscher

**Affiliations:** 1 Department of Population Health Sciences, unit Animals in Science and Society, Faculty of Veterinary Medicine, Utrecht University, Yalelaan 2, 3584 CM Utrecht, The Netherlands; 2 Department of Translational Neuroscience, University Medical Center Utrecht Brain Center, Utrecht University, Utrecht, The Netherlands

**Keywords:** Alcohol consumption, Alcohol use disorder, Aversion, Habit, Motivation, Rats, Loss of control, Individual differences

## Abstract

**Rationale:**

Alcohol use disorder (AUD) is a complex, heterogeneous disorder that only occurs in a minority of alcohol users. Various behavioral constructs, including excessive intake, habit formation, motivation for alcohol and resistance to punishment have been implicated in AUD, but their interrelatedness is unclear.

**Objective:**

The aim of this study was therefore to explore the relation between these AUD-associated behavioral constructs in rats. We hypothesised that a subpopulation of animals could be identified that, based on these measures, display consistent AUD-like behavior.

**Methods:**

Lister Hooded rats (*n* = 47) were characterised for alcohol consumption, habit formation, motivation for alcohol and quinine-adulterated alcohol consumption. The interrelation between these measures was evaluated through correlation and cluster analyses. In addition, addiction severity scores were computed using different combinations of the behavioral measures, to assess the consistency of the AUD-like subpopulation.

**Results:**

We found that the data was uniformly distributed, as there was no significant tendency of the behavioral measures to cluster in the dataset. On the basis of multiple ranked addiction severity scores, five animals (~ 11%) were classified as displaying AUD-like behavior. The composition of the remaining subpopulation of animals with the highest addiction severity score (9 rats; ~ 19%) varied, depending on the combination of measures included.

**Conclusion:**

Consistent AUD-like behavior was detected in a small proportion of alcohol drinking rats. Alcohol consumption, habit formation, motivation for alcohol and punishment resistance contribute in varying degrees to the AUD-like phenotype across the population. These findings emphasise the importance of considering the heterogeneity of AUD-like behavior.

**Supplementary Information:**

The online version contains supplementary material available at 10.1007/s00213-021-06059-4.

## Introduction

Alcohol use disorder (AUD) is a chronic relapsing disorder that is characterised by a lack of control over alcohol use. It is associated with major medical, socioeconomic and legal problems, thereby contributing substantially to the global burden of disease (Connor et al. 2016; Rehm et al. 2009). The pathology of AUD is heterogeneous, and its diagnosis relies on a variety of behavioral criteria such as craving and continued alcohol use despite persistent problems caused by alcohol consumption (American Psychiatric Association 2013). In Europe, approximately 3.7% of the population meets criteria for AUD while more than 75% of the population has been estimated to consume alcohol (World Health Organization 2018). These numbers illustrate that only a minority of all individuals who drink alcohol develop AUD (Anthony et al. 1994). A better understanding of the pathophysiology of AUD, including its underlying neurobehavioral structure, may help to develop improved prevention and treatment strategies for AUD.

In order to elucidate the neural and behavioral mechanisms underlying addictive behavior, animal models have been developed to emulate the diagnostic criteria of AUD (Hopf and Lesscher 2014; Vanderschuren and Ahmed 2013). First, excessive alcohol drinking is a hallmark of AUD that can be studied using limited access choice procedures (Carnicella et al. 2014; Lesscher et al. 2010; Loi et al. 2010; Spoelder et al. 2015). Second, AUD may be driven by automated, habitual behavior, whereby alcohol intake devolves from goal-directed to automated, cue-driven behavior, becoming disconnected from its consequences (Corbit et al. 2012; Dickinson et al. 2002; Everitt and Robbins 2016; Lopez et al. 2014). Outcome devaluation procedures are typically used to dissociate habitual from goal-directed behavior (Barker and Taylor 2014; Dickinson 1985; McKim et al. 2016; Robbins and Costa 2017). Third, the progression from casual to compulsive alcohol use is thought to be accompanied by an increased motivation to obtain alcohol (American Psychiatric Association 2013). In animals, this increased exertion of effort can be measured using progressive ratio (PR) schedules of reinforcement (Hodos 1961; Richardson and Roberts 1996). Fourth, insensitivity to negative consequences is a major characteristic of AUD. Behaviorally, this can be conceptualised as resistance to punishment or aversion, for example by rendering alcohol unsavoury through adulteration with the bitter tastant quinine (Hopf et al. 2010; Hopf and Lesscher 2014; Vanderschuren et al. 2017). Altogether, this range of behavioral tests provides useful tools to study the complexity of human AUD pathology in rodents.

Importantly, preclinical models that mimic the complexity of substance use disorders (SUD), including AUD (Belin-Rauscent et al. 2016), often combine multiple measures of addictive behavior (Ahmed 2012; Belin et al. 2009; Deroche-Gamonet et al. 2004; Domi et al. 2019; Jadhav et al. 2017; Kasanetz et al. 2010; O'Neal et al. 2020; Radke et al. 2017). These models typically also capture the individual variation in behavior, by defining subpopulations portraying a SUD-like phenotype. These subpopulations can then be further investigated for differences in e.g. genetic and behavioral predispositions or to elucidate the underlying neurobiological mechanisms (Augier et al. 2018; Domi et al. 2019; Giuliano et al. 2015; O'Neal et al. 2020; Radwanska and Kaczmarek 2012). However, the interrelations between the different behavioral measures of AUD, for example between individual consumption levels, habitual behavior and loss of control, remain elusive.

Therefore, the aim of this study was to determine how different aspects of AUD-like behavior are related within a sample of Lister Hooded rats. To that end, each rat was characterised for four AUD-like behavioral measures:

1. Voluntary home cage alcohol drinking, determined using an intermittent every-other-day alcohol consumption paradigm,
2. Sensitivity to outcome devaluation after extended operant alcohol self-administration training to examine whether alcohol seeking would progress from a goal-directed to a habitual structure,
3. Motivation for alcohol using a PR schedule of reinforcement and
4. Consumption of quinine-adulterated alcohol as an indicator of aversion-resistant alcohol consumption.

Distributions of all individual measurements were compared to explore if and how these measures are related. Moreover, the consistency of the classification of a subpopulation of rats as AUD-like was investigated using addiction severity scores. We hypothesised that the different AUD-like behavioral measures would be interrelated and that a subgroup of rats could be identified that, based on these behavioral measures, display consistent AUD-like behavior.

## Materials and methods

### Animals

Fifty experimentally naïve adult male Lister Hooded rats (Charles River, Sulzfeld, Germany), weighing 200–250 g (~ 8–10 weeks old) at the start of the experiment, were used in this study. The rats were individually housed in Macrolon type III sawdust bedded cages (42.5 × 26.6 × 18.5 cm) with ad libitum access to tap water and chow (Rat and Mouse Breeder and Grower Expanded-CRM(E), Special Diet Service, UK). A polycarbonate rat tunnel (9 × 9 × 15 cm) and a tissue were provided for cage enrichment. The rats were kept under controlled temperature and humidity conditions (21 ± 2 °C and 50–70% humidity) and on a reversed light/dark cycle (lights off 7.00 AM; lights on 7.00 PM). The rats were acclimatised to the housing conditions for 11 days prior to behavioral testing and they were weighed and handled at least once per week throughout the course of the study. Experimental procedures were approved by the Central Authority for Scientific Procedures on Animals, and were conducted in accordance with Dutch (Wet op de Dierproeven, 2014) and European legislation (Guideline 86/609/EEC; Directive 2010/63/EU).

### Behavioral procedures

**Fig. 1:**
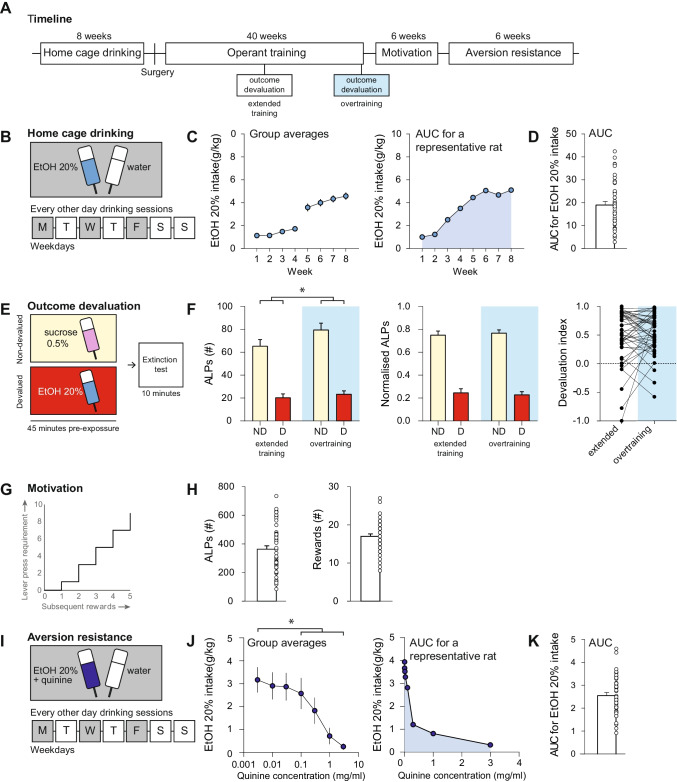
Individual variation in alcohol intake, habit formation, motivation and aversion resistance. **a** Schematic of the experimental design. **b** Schematic of the two-bottle choice intermittent access home cage drinking procedure. The animals initially received 7 h (weeks 1–4) and subsequently 24 h (weeks 5–8) drinking sessions every other day on Mondays, Wednesdays and Fridays. **c** Group average of alcohol intake (g/kg) across 8 weeks of home cage drinking (left panel). For each animal, area under the curve (AUC) values were calculated (blue shaded area) based on their alcohol intake (g/kg) across 8 weeks of home cage drinking (right panel shows a representative animal). **d** Group average (bar) and distribution of individual AUC values for alcohol intake across 8 weeks of home cage drinking. **e** Schematic of the outcome devaluation procedure to test habit formation. All animals were pre-exposed (45 min) to a control solution (sucrose 0.5%, non-devalued) or an alcohol solution (EtOH 20%, devalued). Following pre-exposure, active lever presses (ALPs) were measured for 10 min in the absence of reinforcer delivery (extinction test). **f** Group averages of the number of ALPs during the extinction test for the non-devalued (ND) and devalued (D) condition after extended training and overtraining are shown (blue shaded) (left panel). In addition, normalised ALPs are shown that reflect the distribution of lever pressing for the non-devalued and devalued test after extended training and overtraining (blue shaded) (middle panel). Individual values of the devaluation index after extended training and overtraining (blue shaded) (right panel). **g** Schematic of the progressive ratio schedule of reinforcement to assess motivation. **h** Group average (bar) and distribution of individual values for ALPs averaged across three PR sessions (left panel). Group average (bar) and distribution of individual values for alcohol rewards obtained averaged across three PR sessions (right panel). **i** Schematic of quinine modulation to test aversion resistance. **j** Group average of alcohol intake (g/kg) across drinking sessions with increasing quinine concentrations (left panel). Area under the curve (AUC) values for all animals were calculated (shaded area) based on their alcohol intake (g/kg) across drinking sessions with increasing quinine concentrations (representative animal, right panel). **k** Group average (bar) and distribution of individual AUC values for alcohol intake (g/kg) across drinking sessions with increasing quinine concentrations. Group data are presented as the mean ± SEM. Asterisk (*) denotes significance at a *p* < 0.05 level

Figure 1a provides an overview of the behavioral procedures, which are described in detail below.

#### Intermittent alcohol access (IAA)

Alcohol consumption procedures were performed as previously described (Spoelder et al. 2015, 2017). The rats were exposed to 20% (v/v) alcohol and tap water in a home cage two-bottle choice setup for 3 days a week (Monday, Wednesday, Friday) (Fig. 1a–b). IAA sessions commenced at 9:30 AM and lasted for 7 h (weeks 1–4) or 24 h (week 5 onward). After 8 weeks of IAA, the animals were exposed to alcohol in the home cage on one weekend day, when no training or testing took place, for the remainder of the experiment. Alcohol (99.5%, Klinipath, The Netherlands) was freshly diluted with tap water once per week to a final concentration of 20% (v/v). The position of the bottles was alternated between drinking sessions to avoid the development of a side bias.

#### Operant self-administration

The animals were trained and tested in operant conditioning chambers (29.5 × 24 × 25 cm, Med Associates Inc., USA) in light- and sound-attenuating cubicles equipped with a ventilation fan, controlled by MED-PC IV software (version 4.2). There were two retractable levers (4.8 × 1.9 cm) and a white cue light (28 V, 100 mA) above each lever. One lever was designated as ‘active’, responding on which was reinforced with alcohol access, the other lever was designated as ‘inactive’. The position of active and inactive levers was counterbalanced between rats. A recessed liquid dipper and food receptacle, equipped with an infrared beam for nose poke detection, were situated in between the levers. There was a white house light (28 V, 100 mA) on the opposite side of the box. The floor of the chamber was covered with a metal grid (bars 1.57 cm apart).

#### Fixed ratio and random ratio training

The rats were trained to respond for alcohol during 30-min operant sessions, once daily, 4 days per week. The house light was illuminated throughout the session. The animals were first trained under a fixed ratio (FR) 1 schedule of reinforcement. Pressing the active lever (active lever press; ALP) raised the dipper cup containing an alcohol reward (0.1 ml, 20% v/v). Simultaneously, both levers were retracted and the cue light above the active lever was illuminated. Ten seconds after the animal entered the receptacle, detected by interruption of the infrared light beam, access to alcohol was terminated, the cue light was turned off and the levers were reintroduced, signalling the onset of a new trial. Inactive lever presses were recorded but were without programmed consequences. The alcohol solution was refreshed between sessions.

To determine the development of habitual behavior in our population of rats and the time course of habitual alcohol seeking (after Corbit et al. 2012), we trained our animals to respond for alcohol under a random ratio schedule of reinforcement. The animals were trained twice under a random ratio (RR) 2 schedule of reinforcement during 30-min sessions. The response requirement varied pseudo-randomly between one, two, three or four ALPs for each trial, and was on average two ALPs. Subsequently, all animals were trained under a RR3 schedule of reinforcement during 30-min sessions once daily, 4 days per week. Here, the response requirement varied pseudo-randomly between one, two, three, four, five or six ALPs for each trial, with an average of three ALPs.

#### Outcome devaluation testing

Habit formation was measured through outcome devaluation tests with two conditions (Fig. 1e). In the devalued condition, rats had access to alcohol (EtOH 20%) in the home cage for 45 min. In the non-devalued condition, rats received a 0.5% sucrose solution in the home cage for 45 min. This concentration was chosen to match the volume consumed in the devalued (i.e. alcohol pre-exposure) condition. Animals had ad libitum access to chow during pre-exposure. Immediately after these 45 min, the rats were exposed to a 10-min extinction test to measure responding in the absence of reinforcer delivery. To maintain stable responding on the RR3 schedule of reinforcement, and to allow for within-subjects analysis of habitual alcohol seeking, we trained the rats on a regular RR3 schedule for 30 min after each extinction test. During the extinction tests, both active and inactive levers were presented in the absence of alcohol-associated cues. Responding on the levers was recorded, but was without consequences. Outcome devaluation tests were performed according to a within-subjects Latin square design, with at least one regular 30-min RR3 session day between test days. Sensitivity to outcome devaluation was examined after extended training (50 RR3 sessions) and after overtraining (at least 100 RR3 sessions) (Fig. 1a). After each pair of outcome devaluation tests, the rats were trained in RR3 sessions once daily, 4 days per week.

Two additional outcome devaluation tests were performed as controls. First, after extended training, to control for potential satiation effects of sucrose, an outcome devaluation test was performed with water instead of a sucrose pre-exposure as the non-devalued condition. Second, to rule out that potential sedative effects of alcohol interfered with responding, an outcome devaluation test was performed with a lower alcohol concentration (i.e. EtOH 10%) as the devalued condition in rats with extended training.

#### Progressive ratio schedule of reinforcement

Motivation for alcohol was measured using a progressive ratio (PR) schedule of reinforcement (Fig. 1a). Based on previous studies, in which we found that responding for alcohol is relatively low and the discriminative value of exponential PR schedules between animals is limited, we opted for a linear PR schedule of reinforcement (e.g. Spoelder et al. 2015), where 2 additional ALPs were required for each subsequent alcohol reward (i.e. 1, 3, 5, 7, 9, etc., Fig. 1g). Sessions ended after 100 min and PR performance was considered stable at group level when the average number of ALPs on individual days fell within a 75–125% variability range of the total average of ALPs across those three consecutive days.

#### Quinine modulation of alcohol intake

Aversion resistance was determined by quinine modulation of alcohol intake (Fig. 1a). Alcohol and water consumption were examined using a two-bottle choice setup in the home cage for 24 h every other day, as described. The alcohol solution was adulterated with increasing concentrations of quinine (0, 0.003, 0.01, 0.03, 0.1, 0.3, 1.0, 3.0 mg/ml; Sigma-Aldrich, Germany) (Fig. 1i). Because we observed carry-over effects of high quinine concentrations, possibly reflecting sensitization to the aversive taste of quinine, in previous pilot studies (unpublished findings), we chose to adulterate the alcohol solution with increasing quinine concentrations, rather than in a random order. Each quinine concentration was offered for two consecutive sessions.

### Data analysis and statistics

Data were analysed and visualised using Microsoft Excel, GraphPad Prism (version 8.3.0, GraphPad Software Inc., USA) and RStudio (version 1.2.1335, RStudio Inc., USA). Because three rats did not complete all behavioral procedures, they were excluded from statistical analyses, rendering a final sample of 47 rats. Results are presented as mean ± SEM unless otherwise stated. A significance criterion of *p* < 0.05, two-tailed, was adopted in the statistical analyses.

#### Home cage drinking

The bottles were weighed before and after each drinking session. Fluid intake was calculated by subtracting the bottle weights at the end of every drinking session by the starting weights. Alcohol intake (g/kg) was calculated per rat per session. Weekly averages of alcohol intake throughout intermittent alcohol access were calculated for all animals and the area under the curve (AUC) value was calculated for each animal using GraphPad Prism (version 8.3.0, GraphPad Software Inc., USA). To assess gradual escalation of alcohol consumption as animals progressed to the 24-h sessions, differences in mean alcohol intake (g/kg) between the 7-h (weeks 1–4) and 24-h (weeks 5–8) sessions were compared with a paired samples *t*-test.

#### Outcome devaluation testing

Bottles were weighed before and after pre-exposure. The bottle weights at the end of pre-exposure were subtracted from the starting weights and alcohol fluid intake (ml), alcohol intake (g/kg) or sucrose intake (g/kg) were calculated per rat per session. Outcome devaluation data, i.e. the number of ALPs during the 10-min extinction tests, were analysed by two-way repeated measures ANOVA with *condition* (i.e. non-devalued or devalued) and *timepoint* (i.e. extended training or overtraining) as the within-subjects factors. Similarly, mean ALPs were compared across the two conditions (non-devalued and devalued) for the control outcome devaluation tests with water and a lower alcohol concentration using paired *t*-tests. Moreover, ALPs in the non-devalued and devalued states were normalised to the total number of lever presses (non-devalued + devalued) in each condition. Devaluation indexes were calculated by the following equation: ((#ALP in non-devalued condition)-(#ALP in devalued condition))/((#ALP in non-devalued condition) + (#ALP in devalued condition)). Habit formation was subsequently calculated by subtracting the devaluation index after overtraining from the devaluation index after extended training, such that a positive score indicated greater habit formation.

#### Progressive ratio schedule of reinforcement

The critical parameters for motivation for alcohol were the number of ALPs and the number of rewards obtained, averaged per animal across three consecutive days of stable responding.

#### Quinine modulation of alcohol intake

Bottles were weighed before and after each drinking session. Alcohol intake (g/kg) was calculated as described, and was averaged across two sessions with the same quinine concentration. The alcohol intake data were analysed using a one-way repeated measures ANOVA with *quinine* as the within-subjects factor. Post hoc pairwise Bonferroni comparisons were used to compare alcohol intake at each quinine concentration with non-adulterated alcohol intake. The AUC value for the full quinine dose–response curve was calculated for each animal using GraphPad Prism (version 8.0.1, GraphPad Software Inc., USA).

#### Correlations

To investigate the relations between the different behavioral measurements associated with AUD, the following measurements were selected for correlation analyses:

- Alcohol intake: AUC for alcohol intake (g/kg) across eight consecutive weeks of IAA. A high value indicates high alcohol consumption.
- Habit formation: difference score between the devaluation index after extended training (50 RR3 sessions) and after overtraining (at least 100 RR3 sessions). Values range between − 2 and 2. A positive value (i.e. devaluation index after overtraining is lower than after extended training) indicates a decreased sensitivity to outcome devaluation over time, i.e. habit formation.
- Motivation: average ALPs across three PR sessions. A higher value is indicative of a higher motivation to seek alcohol.
- Aversion resistance: AUC of alcohol intake (g/kg) across increasing concentrations of quinine adulteration. A higher value is indicative of an increased resistance to aversion, i.e. persistent alcohol drinking despite an aversive taste.

One rat was excluded from the correlation analyses as it was a statistical outlier (Z-score > 3.29).

#### Cluster tendency

The cluster tendency in the data was assessed using the Hopkins statistics (Adolfsson et al. 2019; Hopkins and Skellam 1954; Lawson and Jurs 1990). We chose for this method, over unsupervised clustering methods, because unsupervised clustering methods will divide the data into clusters, because that is what they are supposed to do, and will return clusters even if the data does not contain any meaningful clusters. The Hopkins statistics, or H-value, can be considered as a hypothesis test of spatial randomness with the null hypothesis that the dataset is uniformly distributed (i.e. no meaningful clusters) and the alternative hypothesis that the data is not uniformly distributed (i.e. contains meaningful clusters). Highly clusterable datasets will have an H-value that is close to 1 and completely random data will have an H-value that is close to 0.5. Thus, if the H-value < 0.5, then it is unlikely that the dataset contains statistically significant clusters. The analysis was performed using RStudio (version 1.2.1335) and to obtain the Hopkins statistics, we used the *get_clust_tendency()* function from the *factoextrapackage*.

#### Addiction severity scores

Based on alcohol intake, habit formation, motivation and aversion resistance (as described under “Correlations”), an addiction severity score was computed (Belin et al. 2009). Normalisation of each measure was done by subtracting the mean of all animals from the measure for every individual animal that was subsequently divided by the standard deviation of the whole group. This resulted in a score with an average of 0 and a standard deviation of 1 for each measure. In order to determine the consistency of the addiction severity score, and the relative contribution of the different measures on the addiction sensitivity scores, we determined multiple three-criteria addiction severity scores, as a sum of the normalised scores using various compositions of three out of the four behavioral measures, i.e. each time excluding one of the four measures (Fig. 3a). Animals were ranked on their addiction severity score and the highest quartile (*n* = 11) was selected and categorised as the subgroup showing AUD-like behavior. Next, to assess whether animals were consistently categorised as AUD-like, we counted the number of times each animal fell into the highest quartile for the different addiction severity score compositions. The subgroups of animals that were consistently (i.e. belonging to the highest quartile in 4/4 addiction severity score computations) and animals that were never (i.e. belonging to the highest quartile in 0/4 addiction severity score computations) assigned as AUD-like were compared for each of the four behavioral measures using unpaired t-tests.

## Results

### Individual variation in alcohol intake, habit formation, motivation and aversion resistance

#### Home cage drinking

The animals gradually increased their alcohol intake from average levels of 1.4 ± 0.1 g/kg during the 7-h sessions to 4.3 ± 0.3 g/kg during the 24-h sessions (*t*(43) = 13.510, *p* < 0.001) (Fig. 1b–d). Individual animals varied in their levels of alcohol intake, which was most pronounced during the 24-h sessions (Supplementary Fig. 1).

#### Outcome devaluation testing

The data for RR3 training sessions and for the five sessions prior to testing outcome devaluation after extended training (50 RR3 sessions) and overtraining (at least 100 RR3 sessions) are summarized in Supplementary Figs. 2A-D. For outcome devaluation tests, the animals were pre-exposed to alcohol or sucrose, followed by an extinction test (Fig. 1e; Supplementary Fig. 2E). Alcohol pre-exposure reduced responding on the lever associated with alcohol when compared to sucrose pre-exposure, both after extended training and overtraining, demonstrating a significant outcome devaluation effect (*F*_condition_(1,43) = 132.337, *p* < 0.001; *F*_timepoint_ (1,43) = 2.434, *p* = 0.126; *F*_timepoint x condition_ (1,43) = 2.036, *p* = 0.161) (Fig. 1f, left and middle panel). Individual animals showed variation in the development of habit formation over time: for some animals, the devaluation index increased whereas for others the devaluation index decreased or remained comparable (Fig. 1f, right panel).

The effects of outcome devaluation were not dependent on the exposure to sucrose in the non-devalued condition as pre-exposure to water instead of sucrose yielded similar results in that there was a significant outcome devaluation effect (*t*(43) = 11.124, *p* < 0.001; Supplementary Fig. 2F). Furthermore, pre-exposure to a lower concentration of alcohol, to reduce the influence of potential sedative effects of alcohol, resulted in a comparable, significant outcome devaluation effect (*t*(43) = 11.865, *p* < 0.001) (Supplementary Fig. 2G).

#### Progressive ratio schedule of reinforcement

Motivation for alcohol was assessed using a PR schedule of reinforcement (Fig. 1g). On average, the rats made 366 ± 25.7 ALPs and earned 17 ± 0.6 rewards, and individual animals showed considerable variation in their levels of responding (Fig. 1h).

#### Quinine modulation of alcohol intake

Aversion resistance was assessed through quinine modulation of alcohol intake (Fig. 1i). Without quinine, alcohol intake was on average 3.03 ± 0.08 g/kg. As the quinine concentration increased, the animals reduced their alcohol intake from 3.17 ± 0.08 g/kg at a quinine concentration of 0.003 mg/ml to 0.25 ± 0.02 g/kg at a quinine concentration of 3 mg/ml (*F*_Quinine_ (5,200) = 422.781, *p* < 0.001) (Fig. 1j). Post hoc analyses showed that alcohol intake at quinine concentrations of 0.1 mg/ml and higher was significantly lower than alcohol intake without quinine (*p* < 0.001). Individual animals showed considerable variation in their levels of quinine modulated alcohol intake (Fig. 1k).

### Interrelation between the behavioral measures for AUD

To assess the interrelation between the four different behavioral measures (i.e. alcohol intake, habit formation, motivation and aversion resistance), correlation analyses, cluster tendency evaluation and classifications based on addiction severity scores were performed.

#### Correlations and cluster tendency

**Fig. 2:**
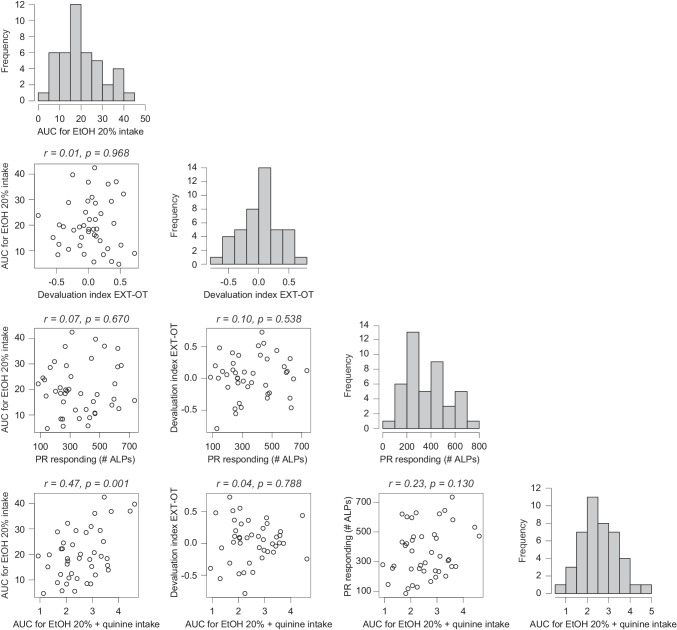
Correlation matrix and frequency plots of alcohol intake, habit formation, motivation and aversion resistance. Alcohol intake is defined as area under the curve (AUC) of weekly home cage alcohol intake (g/kg) during weeks 1–8 of intermittent alcohol access (IAA) (first column). A high AUC value indicates high alcohol consumption. Habit formation is defined as a difference score between the devaluation index after overtraining and after extended training (second row, second column). A habit formation score > 0 indicates a decreased sensitivity to outcome devaluation, i.e. a higher degree of habitual behavior. Motivation for alcohol is defined as the average number of active lever presses made during three progressive ratio (PR) sessions (third row, third column). A higher value is indicative of higher motivation to seek alcohol. Aversion resistance is defined as AUC of alcohol intake (g/kg) across increasing concentrations of quinine adulteration. A higher value is indicative of resistance to aversion, i.e. persistent alcohol drinking despite the aversive taste (fourth row, fourth column). Above every correlation plot, the Pearson correlation coefficient (*r*) and the associated *p*-value are presented

The relations between alcohol intake, habit formation, motivation and aversion resistance were assessed through correlation analyses. The correlation plots are shown in Fig. 2. There was a statistically significant, positive correlation (*r* = 0.47, *p* = 0.001) between alcohol intake during IAA and quinine-adulterated alcohol intake, indicating that rats that consumed more alcohol were likely to consume more alcohol during quinine adulteration (Fig. 2, fourth row, first column). Other correlations were weak (i.e. − 0.25 < *r* < 0.25) and not significant (*p* ≥ 0.130). The tendency to cluster in the complete dataset was evaluated using the Hopkins statistic. The H-value was 0.475, which indicates that overall, the data set was uniformly distributed rather than clustered into a meaningful grouping structure.

#### Classification of AUD-like animals based on three-criterion addiction severity scores

**Fig. 3:**
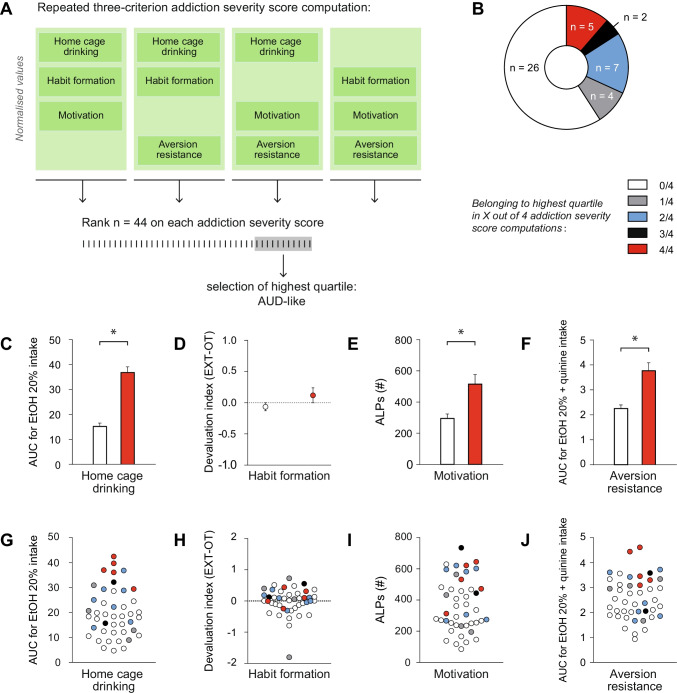
Addiction severity scores based on combinations of alcohol intake, habit formation, motivation and aversion resistance. The addiction severity scores were computed and the highest quartile (*n* = 11) was identified as rats showing typical AUD-like behavior. When any of the four measures was excluded from the computation, the composition of rats in the highest quartile changed. **a** Schematic of the repeated three-criterion addiction severity score computation procedure. **b** Division of animals based on individual rats belonging to the highest quartile in the addiction severity score computations. Individual animals were labelled according to the frequency in which they were assigned to the highest quartile, i.e. never (0/4; white), once (1/4; grey), twice (2/4; blue), three times (3/4; black), consistently (4/4; red). **c** Average area under the curve (AUC) values for alcohol intake (g/kg) across 8 weeks of home cage drinking, **d** Average of the difference score between the devaluation indexes after extended training versus overtraining, **e** Average active lever presses (ALPs) averaged across three progressive ratio (PR) sessions and **f** Average AUC values for alcohol intake (g/kg) across drinking sessions with increasing quinine concentrations for animals that never fell into the highest quartile (*n* = 26; white) and for animals that always fell into the highest quartile (*n* = 5; red). **g** Distributions of individual AUC values for alcohol intake (g/kg) across 8 weeks of home cage drinking. **h** Individual values of the difference score between the devaluation indexes after extended training versus overtraining. **i** Distributions of individual values for ALPs averaged across three PR sessions. **j** Distributions of individual AUC values for alcohol intake (g/kg) across drinking sessions with increasing quinine concentrations. Individual animals were labelled on the basis of selection in the highest quartile, i.e. never (0/4; white), once (1/4; grey), twice (2/4; blue), three times (3/4; black), consistently (4/4; red) AUD-like. Group data are presented as the mean ± SEM. Asterisk (*) denotes significance at a *p* < 0.05 level

Based on alcohol intake, habit formation, motivation and aversion resistance, we also computed addiction severity scores (Belin et al. 2009). Specifically, multiple three-criteria addiction severity scores were calculated by consecutively excluding one of the measures, thus resulting in four three-criteria addiction severity scores. Next, for each computation, animals with the highest addiction severity scores were identified, i.e. the rats that fell within the highest quartile (*n* = 11), and were labelled as showing AUD-like behavior (Fig. 3a). Subsequently, the consistency of the classification of AUD-like animals among the various three-criterion addiction severity score compositions was assessed (Fig. 3b). A subgroup of five animals was consistently classified as AUD-like (4/4; *n* = 5). The majority of rats was never classified as AUD-like (0/4; *n* = 26), independent of the composition of the three-criterion addiction severity score. The remaining animals were classified as AUD-like once (1/4; *n* = 4), twice (2/4; *n* = 5) or three out of four times (3/4; *n* = 2). Thus, depending on the combination of behavioral measures, different animals were classified as AUD-like, while a small group of animals displayed consistent AUD-like behavior.

The subgroup of animals that was consistently classified as AUD-like was compared to the subgroup of animals that was never classified as AUD-like. The consistent AUD-like animals showed significantly higher levels of alcohol intake (Fig. 3c), PR responding (Fig. 3e) and higher levels of quinine-adulterated alcohol intake (Fig. 3f) compared to the animals that were never classified as AUD-like (alcohol intake: *t*(29) = 7.173, *p* < 0.001; PR responding: *t*(29) = 3.316, *p* = 0.0025; quinine-adulterated alcohol intake: *t*(29) = 4.581, *p* < 0.001). However, habit formation was comparable between the subgroups (Fig. 3d, t(29) = 1.166, *p* = 0.253). These findings confirm that the rats that were consistently classified as AUD-like consumed more alcohol, showed higher motivation and were more aversion-resistant than the rats that were never classified as AUD-like.

Although subgroups could be identified based on the addiction severity scores, there was considerable individual variation for each of the four behavioral parameters (Fig. 3g–j). Whether or not an animal was consistently assigned as AUD-like (i.e. within the highest quartile in 4/4 computations) matched most clearly with the level of alcohol intake, since all five animals of the consistent AUD-like subgroup fell into the highest quartile for alcohol intake (Supplementary Table 1). Similarly, the animals of the consistent AUD-like subgroup generally showed high levels of quinine-adulterated alcohol intake as most of them were in the highest quartile when ranked based on quinine-adulterated alcohol intake. However, scores of consistently assigned AUD-like animals and never assigned as AUD-like animals overlapped, most prominently for the habit formation and motivation parameters. When also considering the intermediate groups, this overlap in scores on each of the behavioral measures was even more pronounced. Thus, while a subgroup could be identified that consistently showed AUD-like behavior, there was considerable overlap with the other animals due to the widespread individual variation in each of the four behaviors.

## Discussion

The present study explored the relationship between four behavioral measures for AUD-like behavior in rats: alcohol intake, habit formation, motivation, and aversion resistance. Within the population of rats in this study, we report considerable individual variation for all AUD-like behaviors, with a correlation between alcohol consumption and aversion-resistant alcohol consumption across the population. Overall, the selection of animals with a high addiction severity score varied substantially, depending on which of the four measures were included. This means that being designated as AUD-like based on one form of alcohol-directed behavior is not necessarily indicative of a high score for the other AUD behaviors. However, a group of five animals consistently displayed AUD-like behavior.

The purpose of the present study was to assess whether four aspects of AUD-like behavior i.e. voluntary alcohol consumption, motivation for alcohol, aversion-resistant alcohol consumption and habitual alcohol seeking are interrelated. To that aim, a combination of analyses was used. Correlation analyses revealed that the strongest association was observed between alcohol consumption and aversion-resistant alcohol consumption: animals that consumed the highest levels of alcohol exhibited more resistance to quinine adulteration. This is in line with previous studies that reported greater aversion resistance in quinine-adulterated alcohol intake in rats with a high alcohol drinking phenotype (Hopf et al. 2010; Spoelder et al. 2015). None of the other correlations reached statistical significance. It should be noted that, as a result of the longitudinal setup of the current experiments, some of the behavioral assessments were separated in time by months, e.g. alcohol intake during IAA and PR responding, which may explain the absence of a significant correlation. Indeed, AUD-associated behaviors are not static, but may rather can change over time during alcohol exposure (Jadhav et al. 2017; Spoelder et al. 2017). Therefore, we cannot exclude the possibility that some measures would have shown a different association had they been performed closer to one another in time. That said, alcohol consumption levels were comparable to previous studies that reported blood alcohol levels ranging between 20 and 40 mg/kg 30 min after onset of the drinking session (Cippitelli et al. 2012; Loi et al. 2010; Sabino et al. 2013; Simms et al. 2008; Spoelder et al. 2015) and remained stable throughout the study, suggesting that tolerance to alcohol did not emerge and affect the data. Moreover, the IAA period and the quinine-adulterated alcohol exposure were most distant in time, yet showed the strongest correlation. In fact, a recent study showed that resistance to punishment initially did not correlate with responding for alcohol, but did so after > 75 alcohol training sessions (Jadhav et al. 2017). In this same study, the correlation between excessive motivation for alcohol and responding for alcohol increased over time (Jadhav et al. 2017). Thus, the separation in time of the behavioral measurements is not likely to be the sole explanation for the absence of correlations between them. In addition, although we cannot exclude that the order of the behavioral tests may have influenced the correlations, apart from the potential effects of prolonged alcohol exposure, we have no reason to assume that the measurements affect one another. The only exception perhaps is the quinine adulteration of the alcohol solution that may cause a long-term devaluation of the alcohol solution, which is why aversion resistance was assessed as the final measurement. In fact, the weak (and non-significant) correlations between most of our parameters are in line with highly variable relationships between different metrics in a recent study on heroin addiction–like behavior (O'Neal et al. 2020), and suggest that the measures reflect distinct aspects of AUD-like behavior.

No significant tendency to cluster was detected for our data, suggesting that on the basis of the four behavioral components in this study, no distinct AUD-like subpopulation could be identified whose scores are clearly dissociable from the others. However, the four behavioral measures were continuous variables and assumed to be normally distributed. Consequently, the fact that there is no tendency to cluster in the data does not exclude the possibility that there might be individual animals that score consistently high or low on the different measures. Therefore, further analysis of the data was performed using addiction severity scores. The addiction severity score was computed repeatedly, each time excluding one of the four variables, to assess variability in the composition of the group that exhibits AUD-like behavior. Five animals (~ 11% of our sample) were consistently classified as displaying AUD-like behaviors across all addiction severity score computations. The rats that displayed consistent AUD-like behavior consumed higher levels of alcohol, showed higher motivation for alcohol, and portrayed more aversion resistance, compared to rats that were classified as displaying the least AUD-like behavior, confirming the overall AUD-like phenotype of these animals. Importantly, these results are comparable to humans, where only a minority of alcohol consumers develops AUD (Anthony et al. 1994). The composition of the remaining animals that were attributed to the highest quartiles of the addiction severity score varied substantially, depending on which of the four measures were included. These findings resonate the lack of correlation between the different AUD-like measures in this study, i.e. for the majority of the animals, individual alcohol intake levels for instance do not consistently predict the motivation for alcohol. This indicates that a significant proportion of animals score high in one or more categories, but low in others, suggesting a substantial individual heterogeneity in the neurobehavioral constructs underlying AUD-like behavior.

One limitation of the study is that overtraining in our operant self-administration task did not result in habit development for alcohol seeking at a group level. The degree of sensitivity to outcome devaluation was similar after an extended period of training (50 RR3 sessions) and after overtraining (at least 100 RR3 sessions), suggesting that responding for alcohol remained goal-directed at both timepoints. The absence of habit detection is in contrast to studies that reported reduced sensitivity to changes in outcome value after a prolonged training period for alcohol, cocaine and nicotine (Clemens et al. 2014; Corbit et al. 2012; LeBlanc et al. 2013; Zapata et al. 2010). However, conflicting results have also been reported (Halbout et al. 2016; Samson et al. 2004). Several studies suggest that discriminative cues, i.e. the presentation of a tone, cue light or even the insertion of the levers, may facilitate the expression of habitual behaviors (Thrailkill et al. 2021; Vandaele et al. 2017). Therefore, the conflicting results with regards to the emergence of habitual responding for rewards may be related to the strength or weakness of the cues used. In humans, studies that demonstrate a decrease in sensitivity to devaluation as a function of behavioral repetition are scarce. Some studies reported overreliance on habit learning in AUD or a reduced sensitivity to devaluation after extensive training (Sjoerds et al. 2013; Tricomi et al. 2009), but recent studies report no evidence for habit formation in human subjects after prolonged training (de Wit et al. 2018; Hogarth et al. 2019; Hogarth 2020; Luijten et al. 2020). It is also notable that habit formation was the only parameter that did not significantly differ between animals that showed most and least AUD-like behavior (Fig. 3d). This suggests that habit formation played only a minor role in AUD-like behavior in the present study, although the absence of habit development at a group level suggests that these data should be interpreted with caution. A further limitation to this study is the choice for a limited number of AUD-like behaviors. We did for instance not consider other aspects of loss of control, i.e. footshock-resistant responding for alcohol (Lesscher and Vanderschuren 2012; Spoelder et al. 2017; Vanderschuren et al. 2017), nor did we do extensive behavioral analyses to evaluate the development of withdrawal signs.

## Conclusion

In this study, the relation between various behavioral constructs that have been implicated in AUD was explored in a sample of rats. Apart from a small group of animals that consistently displayed AUD-like behavior, we observed considerable individual variation for all AUD-like behaviors, reflected by substantial variation in the selection of animals with a high addiction sensitivity score, depending on the measures included. Our findings emphasise the importance of considering the heterogeneity in relative contribution of behavioral constructs driving AUD-like behavior. First, this heterogeneity may have implications for pinpointing underlying neural substrates and predispositions for AUD using preclinical studies. Second, taking this heterogeneity into account might facilitate the translation to human psychopathology, as AUD in humans is also considered a very heterogeneous pathology in terms of symptom dimensions, disease severity, treatment response and comorbidities (Schuckit 2006).

## Supplementary Information

Below is the link to the electronic supplementary material.

Supplementary Figure 1. Home cage alcohol intake. A. Group average (bar) and distribution of individual values for alcohol intake averaged across all 7-hour sessions. B. Group average (bar) and distribution of individual values for alcohol intake averaged across all 24-hour sessions. Asterisk (*) denotes significance at a p < 0.05 level as compared to Supplementary Figure 1A. Group data are presented as the mean ± SEM.
Supplementary file1 (PDF 172 KB)

Supplementary Figure 2. Habit formation. A. Group average of the RR3 training response rate (active lever presses (blue), inactive lever presses (grey)) five days prior to outcome devaluation in the extended training phase. B. Group average of the RR3 training response rate (active lever presses (blue), inactive lever presses (grey)) five days prior to outcome devaluation in the overtraining phase. C. Group average of the rewards obtained during RR3 training five days prior to outcome devaluation in the extended training phase. D. Group average of the rewards obtained during RR3 training five days prior to outcome devaluation in the overtraining phase. E. Schematic of the pre-exposure procedure (left panel). Group average of solution intake during pre-exposure for the non-devalued (sucrose 0.5%, non-devalued, pink) or devalued (EtOH 20%, devalued, blue) condition for the extended training and overtraining (blue shaded) (right panel). Average alcohol intake (g/kg) is indicated below the respective devalued conditions. F. Schematic of the pre-exposure procedure with water instead of 0.5% sucrose in the non-devalued condition (left panel). All animals were pre-exposed (45 minutes) to a control solution (water, non-devalued, white) or an alcohol solution (EtOH 20%, devalued, blue). Group average of solution intake during pre-exposure for the non-devalued (water, non-devalued, white) or devalued (EtOH 20%, devalued, blue) (middle panel). Average intake (g/kg) of alcohol is indicated below the devalued condition. Group averages of active lever presses (ALPs) made during the extinction test for the non-devalued (ND) and devalued (D) condition (right panel). G. Schematic of the pre-exposure procedure with a lower alcohol concentration (EtOH 10%, left panel). All animals were pre-exposed (45 minutes) to a control solution (sucrose 0.5%, non-devalued, pink) or an alcohol solution (EtOH 10%, devalued, blue). Group average of solution intake during pre-exposure for the non-devalued (water, non-devalued, white) or devalued (EtOH 10%, devalued, blue) (middle panel). Average intake (g/kg) of alcohol is indicated below the devalued condition. Group averages of ALPs made during the extinction test for the non-devalued (ND) and devalued (D) condition (right panel). Group data are presented as the mean ± SEM. Asterisk (*) denotes significance at a p < 0.05 level.
Supplementary file2 (PDF 216 KB)

Supplementary file3 (DOCX 20 KB)
