# Supplementary figures and images for: On the interrelation between alcohol addiction–like behaviors in rats
Source: Psychopharmacology (Berl). 2022 Jan 12;239(4):1115–28. doi: 10.1007/s00213-021-06059-4 (PMC8986720; doi:10.1007/s00213-021-06059-4)

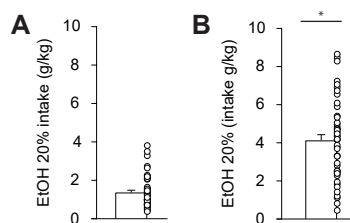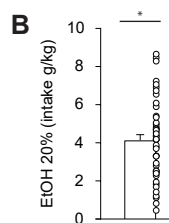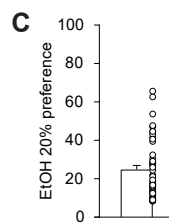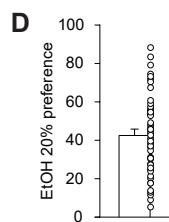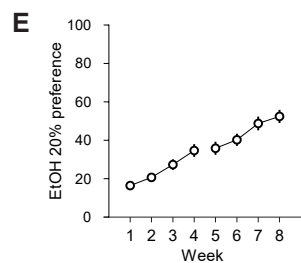

Supplement: Supplementary file 1 — Supplementary Figure 1. Home cage alcohol intake. A. Group average (bar) and distribution of individual values for alcohol intake averaged across all 7-hour sessions. B. Group average (bar) and distribution of individual values for alcohol intake averaged across all 24-hour sessions. Asterisk (*) denotes significance at a p < 0.05 level as compared to Supplementary Figure 1A. Group data are presented as the mean ± SEM. Supplementary file1 (PDF 172 KB) [file 213_2021_6059_MOESM1_ESM.pdf]

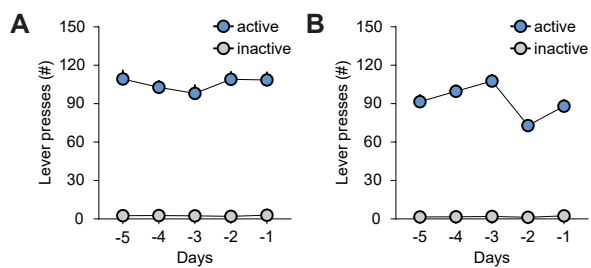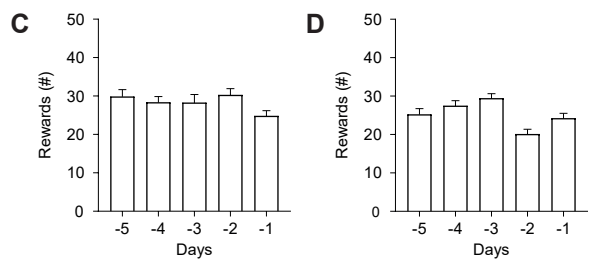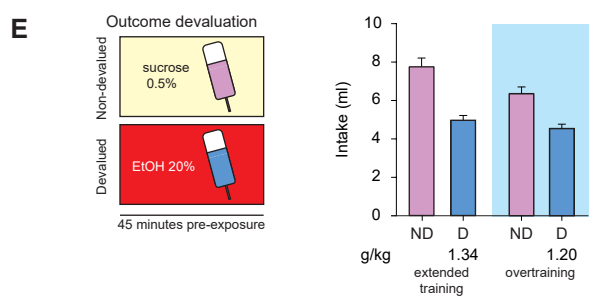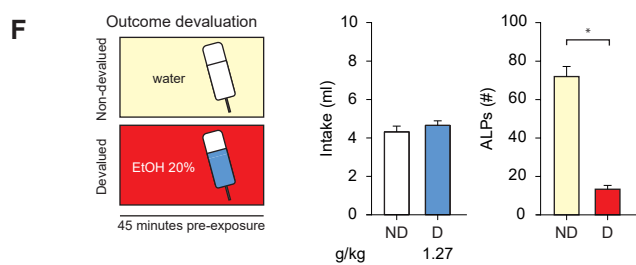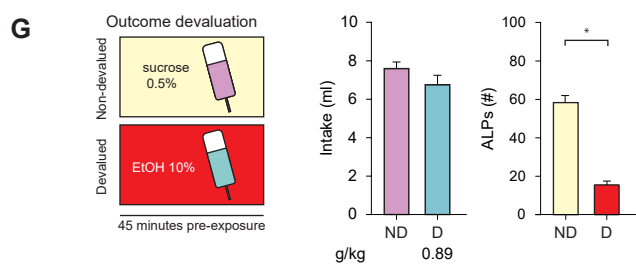

Supplement: Supplementary file 2 — Supplementary Figure 2. Habit formation. A. Group average of the RR3 training response rate (active lever presses (blue), inactive lever presses (grey)) five days prior to outcome devaluation in the extended training phase. B. Group average of the RR3 training response rate (active lever presses (blue), inactive lever presses (grey)) five days prior to outcome devaluation in the overtraining phase. C. Group average of the rewards obtained during RR3 training five days prior to outcome devaluation in the extended training phase. D. Group average of the rewards obtained during RR3 training five days prior to outcome devaluation in the overtraining phase. E. Schematic of the pre-exposure procedure (left panel). Group average of solution intake during pre-exposure for the non-devalued (sucrose 0.5%, non-devalued, pink) or devalued (EtOH 20%, devalued, blue) condition for the extended training and overtraining (blue shaded) (right panel). Average alcohol intake (g/kg) is indicated below the respective devalued conditions. F. Schematic of the pre-exposure procedure with water instead of 0.5% sucrose in the non-devalued condition (left panel). All animals were pre-exposed (45 minutes) to a control solution (water, non-devalued, white) or an alcohol solution (EtOH 20%, devalued, blue). Group average of solution intake during pre-exposure for the non-devalued (water, non-devalued, white) or devalued (EtOH 20%, devalued, blue) (middle panel). Average intake (g/kg) of alcohol is indicated below the devalued condition. Group averages of active lever presses (ALPs) made during the extinction test for the non-devalued (ND) and devalued (D) condition (right panel). G. Schematic of the pre-exposure procedure with a lower alcohol concentration (EtOH 10%, left panel). All animals were pre-exposed (45 minutes) to a control solution (sucrose 0.5%, non-devalued, pink) or an alcohol solution (EtOH 10%, devalued, blue). Group average of solution intake during pre-exposure for the non-deva [file 213_2021_6059_MOESM2_ESM.pdf]
