# Supplementary material for: On the interrelation between alcohol addiction–like behaviors in rats
Source: Psychopharmacology (Berl). 2022 Jan 12;239(4):1115–28. doi: 10.1007/s00213-021-06059-4 (PMC8986720; doi:10.1007/s00213-021-06059-4)
Supplement: Supplementary file 3 — Supplementary file3 (DOCX 20 KB) [file 213_2021_6059_MOESM3_ESM.docx]

**Supplementary Table 1. Ranking of the population on each behavioral measure.** All animals were ranked on their scores on alcohol intake, habit formation, motivation, and aversion resistance. 1 refers to the highest score within the population, 44 refers to the lowest score within the population.

|  | *Ranking place* | *Ranking place* | *Ranking place* | *Ranking place* |
| --- | --- | --- | --- | --- |
| animal ID | **Alcohol intake** | **Habit formation** | **Motivation** | **Aversion resistance** |
| 3 | 2 | 35 | 12 | 1 |
| 14 | 3 | 5 | 10 | 2 |
| 35 | 8 | 27 | 2 | 11 |
| 40 | 1 | 18 | 23 | 7 |
| 49 | 5 | 9 | 4 | 13 |
| 6 | 28 | 15 | 1 | 5 |
| 42 | 6 | 2 | 16 | 30 |
| 15 | 12 | 28 | 24 | 8 |
| 16 | 27 | 19 | 7 | 32 |
| 18 | 31 | 13 | 8 | 4 |
| 26 | 15 | 17 | 6 | 35 |
| 34 | 9 | 8 | 27 | 24 |
| 38 | 4 | 26 | 30 | 3 |
| 44 | 10 | 37 | 5 | 40 |
| 10 | 38 | 1 | 17 | 39 |
| 21 | 17 | 6 | 36 | 14 |
| 32 | 32 | 44 | 9 | 9 |
| 37 | 7 | 22 | 38 | 16 |
| 1 | 44 | 4 | 40 | 43 |
| 2 | 25 | 34 | 11 | 18 |
| 4 | 43 | 20 | 32 | 25 |
| 5 | 18 | 39 | 25 | 21 |
| 7 | 13 | 12 | 43 | 34 |
| 8 | 24 | 24 | 29 | 6 |
| 9 | 11 | 16 | 39 | 20 |
| 11 | 39 | 29 | 21 | 19 |
| 12 | 30 | 42 | 31 | 42 |
| 13 | 37 | 36 | 13 | 23 |
| 17 | 42 | 7 | 18 | 36 |
| 20 | 41 | 41 | 33 | 33 |
| 22 | 40 | 11 | 34 | 17 |
| 24 | 16 | 23 | 44 | 37 |
| 25 | 22 | 14 | 35 | 22 |
| 27 | 35 | 32 | 22 | 12 |
| 29 | 29 | 31 | 15 | 15 |
| 30 | 20 | 38 | 26 | 44 |
| 31 | 33 | 40 | 3 | 27 |
| 33 | 26 | 25 | 41 | 29 |
| 36 | 34 | 3 | 19 | 38 |
| 41 | 19 | 30 | 28 | 41 |
| 43 | 36 | 10 | 14 | 28 |
| 46 | 21 | 33 | 37 | 10 |
| 47 | 23 | 21 | 20 | 31 |
| 50 | 14 | 43 | 42 | 26 |
